# The Pasting and Gel Textural Properties of Corn Starch in Glucose, Fructose and Maltose Syrup

**DOI:** 10.1371/journal.pone.0095862

**Published:** 2014-04-22

**Authors:** Qingjie Sun, Yan Xing, Chao Qiu, Liu Xiong

**Affiliations:** 1 College of Food Science and Engineering, Qingdao Agricultural University, Qingdao, Shandong Province, China; 2 Department of Grain Reserve and Control, Lishui Bureau of Commerce, Nanjing, Jiangsu Province, China; China Agricultural University, China

## Abstract

The pasting and gel textural properties of corn starch in syrup at different concentrations were investigated by Rapid Visco Analyzer (RVA) and Texture profile analysis (TPA) tests. The results showed that the pasting temperatures of corn starch greatly increased, especially at higher sugar concentration. Increasing concentration of syrup caused an increase in peak, trough and final viscosity of corn starch. Peak viscosity and the disintegration rate of starch increased in the following order: fructose syrup> maltose syrup> glucose syrup. Increasing syrup concentration to 13%, 25% and 50% resulted in a lower retrogradation rate than the control. When the maltose syrup concentration increased to 50%, the retrogradation rate decreased to 14.30% from 33.38%. The highest hardness was observed when the syrup concentration was 25%. There was a particular low hardness when the concentration of syrup was 50%. The springiness of starch gels in syrup was similar at different concentrations.

## Introduction

Corn starch is a valuable ingredient to the food industry, being widely used as a thickener, gelling agent, bulking agent and water retention agent [1]. Pasting properties, gelatinization and subsequent textural properties of starch are key functional properties that determine many applications of starch in the food industry [2]. Native starch undergoes various physicochemical changes during thermal processing. Specifically, when heated in water, starch granules swell, followed by disruption of their crystalline structures [3]. The rising viscosity has been ascribed to the swelling of the starch granules as they absorb water until they burst. The viscosity maximum is reached when the granules are fully swelled, and the subsequent decrease results from the less rigid arrangement of the individual molecules released by granules rupture [4]. Subsequently, gelatinized starch molecules are re-associated in an ordered structure. During this heating and cooling process, the texture of the resultant starch pastes changes, thereby forming viscoelastic gels. However, the structure and property of starch are highly dependent on its sources and also varies under different processing conditions [5].

Recently, some studies on the pasting properties, gelatinization and textural properties of starch–sugar composites have been reported. Peak viscosity, trough viscosity and final viscosity of wheat and potato starches increased as the concentration of sucrose, glucose, and glycerol increased. For both starches peak viscosity increased in the following order: glucose> sucrose>glycerol [2]. Sharma et al. [6] found that the pasting temperature increased with the increase of the sugar concentration in cassava starch–water system, while peak viscosity and breakdown viscosity decreased with the increase of the sugar concentration. Perry et al. [7] reported that the addition of sugars and other polyols to starch-water systems elevates the starch gelatinization temperature. Sugars delay starch gelatinization by increasing the gelatinization temperature [8]. Gelatinization temperature of wheat and potato starches were increased by sucrose, glucose and glycerol in the order of sucrose>glucose>glycerol. Gel hardness of wheat starch was increased following the order glucose>sucrose>glycerol [2].

Syrup is a kind of sticky solution containing high levels of sugar. Syrup and sugar is important ingredient in many starch based foods. Saleem et al. [9] demonstrated material properties of semi-sweet biscuits and biscuits contained 1.3% glucose and 21% sugar. Secchi et al. [10] studied the shelf life of Amaretti cookies and two batches of Amaretti cookies contained 18% and 22% sugar. A gummy confection consists of high proportions of sucrose and syrup (60%), combined with starch [11].

Currently, in most studies, the effect of sugar on starch gelatinization is investigated by using differential scanning calorimetry. The present studies on pasting properties of starch are contradictory. For example, the peak viscosity and breakdown viscosity of cassava starch decreased with increase in the sugar concentration [6], but peak viscosity and breakdown point of wheat and potato starches increased as the concentration of sucrose, glucose, and glycerol increased [2]. However, no systematic studies have been reported on textural properties of starch in sugar or syrup. Therefore the purpose of this study was to investigate the influence of corn starch addition on glucose, fructose and maltose syrup at different concentrations (from 0% to 50%). The pasting and textural properties were analyzed in order to better understand the interaction between starch and syrup. A Rapid Visco Analyzer (RVA) has been generally employed to investigate the pasting properties of starch by monitoring its viscosity during heating and cooling. In addition, the textural analysis of starch gel has been carried out using a texture profile analysis (TPA). Based on this study, we can systematically understand the pasting and textural properties of corn starch in syrup and the addition of syrup to corn starch in suitable proportions can be a feasible alternative to formulation of starch based foods.

## Materials and Methods

### Materials

Corn starch (amylose content 26.33%) was from Shandong Zhucheng starch company, China. Glucose, fructose and maltose syrup (moisture content 25%) were purchased from Shandong Luzhou food Co., Ltd., China.

**Table 1:** RVA values of corn starch in glucose syrup at different concentrations.

Glucose syrupconcentr-ations/%	Pastingtemperature/°C	Peakviscosity/RVU	Troughviscosity/RVU	Finalviscosity/RVU	Breakdown/RVU	Disintegrati-onrate/%	Setback/RVU	Retrogr-adationrate/%
0	77.20±0.14a	235.79±2.65a	170.04±1.36a	248.75±1.06a	65.75±4.01c	27.88±1.39d	78.71±2.42ab	33.38±0.65d
1	77.30±0.28a	244.17±2.83b	173.13±3.12a	261.92±0.47a	71.04±2.95c	29.09±1.10d	88.79±2.65b	36.36±0.66d
3	77.10±0.14a	253.29±1.12c	182.04±1.00a	266.00±0.94a	71.25±2.12c	28.13±0.71d	83.96±0.06ab	33.15±0.12d
13	78.60±0.14b	320.92±0.71d	245.83±1.89b	331.42±0.01b	75.09±1.59c	23.40±0.76c	85.59±6.90ab	26.67±2.40c
25	82.60±0.28c	413.71±0.41e	360.58±0.71c	425.21±0.65c	53.13±0.29b	12.84±0.08b	64.63±1.36a	15.62±0.34a
50	95.05±0.07d	532.49±2.06f	534.17±0.79d	636.83±1.74d	−1.68±0.06a	−0.32±0.02a	102.66±2.86c	19.28±1.00b

All data represent the mean of three determinations.

Mean±standard deviation.

Means with the same letter in each column are not significantly different (p<0.05).

**Table 2:** RVA values of corn starch in fructose syrup at different concentrations.

Fructose syrupconcentrations/%	PastingTemperature/°C	Peakviscosity/RVU	Troughviscosity/RVU	Finalviscosity/RVU	Breakdown/RVU	Disintegrati-onrate/%	Setback/RVU	Retrogr-adation rate/%
0	77.20±0.14a	235.79±2.65a	170.04±1.36a	248.75±1.06a	65.75±4.01b	27.88±1.39b	78.71±2.42b	33.38±0.65c
1	76.90±0.57a	273.42±0.94b	188.25±4.60b	285.79±3.01b	85.17±3.65c	31.15±1.44bc	97.54±1.59c	35.67±0.70c
3	76.90±0.07a	286.46±1.12c	195.38±4.54b	296.42±0.24c	93.08±5.66c	31.80±1.85bc	103.04±4.77c	35.27±1.53c
13	78.40±0.57b	403.38±0.88d	270.79±4.89c	371.29±0.53d	138.59±3.01d	32.87±1.07bc	106.50±4.36c	24.91±1.14b
25	82.00±0.57c	585.17±0.82e	409.25±9.55d	554.46±0.06e	175.92±2.72e	30.06±1.53bc	145.21±2.49d	24.82±1.66b
50	95.40±0.14d	846.49±2.30f	807.67±0.82e	807.75±0.71f	38.82±2.12a	4.59±0.36a	0.08±0.12a	0.00±0.00a

All data represent the mean of three determinations.

Mean± standard deviation.

Means with the same letter in each column are not significantly different (p<0.05).

### Methods

#### Pasting properties of corn starch

The pasting properties of the starch were evaluated with the Rapid Visco Analyzer (RVA-4, Newport Scientific, Warriewood, Australia). Corn starch (3.0 g, 14 g/100 g moisture basis) was weighed directly in the RVA canister and syrup was added to obtain a sample weight of 28.0 g. The concentration of syrup was 0%, 1%, 3%, 13%, 25% and 50%. A programmed heating and cooling cycle was used, where the samples were held at 50°C for 1 min, heated to 95°C at 12°C/min, held at 95°C for 2.7 min, before cooling from 95 to 50°C at 12°C/min and holding at 50°C for 2 min. Parameters recorded were pasting temperature, peak viscosity, trough viscosity (minimum viscosity at 95°C), final viscosity (viscosity at 50°C), breakdown viscosity (peak viscosity - trough viscosity) and setback viscosity (final viscosity - trough viscosity) [12]. All measurements were replicated thrice. All tests were replicated three times.

#### Textural properties of starch gels

The starch prepared in the RVA was poured into small aluminum canisters and stored at 4°C to cause gelation. The gels formed in the canisters were evaluated for their textural properties by texture profile analysis (TPA) using the TA/XT2 texture analyzer (Stable MicroSystems, Surrey, England). Each canister was placed upright on the metal plate and the gel was compressed at a speed of 0.5 mm/s to a distance of 10 mm with a cylindrical plunger (diameter = 5 mm). The compression was repeated twice to generate a force–time curve from which hardness (height of first peak) and springiness (ratio between recovered height after the first compression and the original gel height) was determined. Five repeated measurements were performed for each sample and their average was taken [13].

#### Statistical analysis

All experiments were conducted at least in triplicate, for which mean values and standard errors were determined. Also, experimental data were analyzed using Analysis of Variance (ANOVA), and expressed as mean values ± standard deviations. Differences were considered at significant level of 95% (P<0.05). Pearson’s correlation coefficients among parameters were calculated using SPSS v17.0 software.

## Results and Discussion

### Pasting Properties of Corn Starch

Pasting results of corn starch in syrup at different concentrations were presented (Table 1 to Table 3). From Table 1 to Table 3, we could see that there was no significant difference in pasting temperatures when the concentration of syrup was 0%, 1% and 3%. But when the concentration of syrup was 13%, 25% and 50%, pasting temperatures of corn starch increased significantly. Similar results were reported by Sharma et al. [6]. The high pasting temperature of starch indicated its higher resistance towards swelling [14]. The competition between sucrose and starch, through association with available water molecules, leads to a decrease in water activity in the mix and restrains the swelling of starch [15]. The increasing concentration of syrup caused an increase in peak viscosity, trough viscosity and final viscosity of corn starch. For instance, peak viscosity of corn starch in fructose syrup at 25% concentration was more than twice than that at 0% concentration. Peak viscosity of corn starch increased in the following order: fructose syrup> maltose syrup> glucose syrup. Comparing the three kinds of syrup, we could found corn starch in glucose syrup had the lowest peak viscosity, trough viscosity and final viscosity. Maybe because it was more difficult for starch granules to move than in water and could cohere together better, leading to close packing concentration of swollen corn starch granules. Similar results were reported by Richardson et al. [16]. Also in syrup, the hydration of corn starch granules was inhibited. Similar results were reported by Zhou et al. [17]. Previously, Mantzari et al. [18] demonstrated that the increase in viscosity of starch examined was proportional to the sorbitol content increase. This could be attributed to the fact that the external surface of the amylose helices bears hydroxyl groups which could interact with the hydroxyl groups of sorbitol to form hydrogen bridges. Sharma et al. [6] reported that the peak viscosity of cassava starch decreased with increase in the sugar concentration. The fact that the results of this study were opposite to those of the former study may be because the raw materials were different.

**Table 3:** RVA values of corn starch in maltose syrup at different concentrations.

Maltose syrupconcentrations/%	Pastingtemperature/°C	Peakviscosity/RVU	Troughviscosity/RVU	Finalviscosity/RVU	Breakdown/RVU	Disintegrati-onrate/%	Setback/RVU	Retrograd-ationrate/%
0	77.20±0.14b	235.79±2.65a	170.04±1.36a	248.75±1.06a	65.75±4.01ab	27.88±1.39cd	78.71±2.42a	33.38±0.65d
1	76.25±0.21a	274.17±0.82b	189.04±1.00b	287.75±1.06b	85.13±0.71c	31.05±1.52d	98.71±2.06b	36.00±0.64e
3	76.80±0.57ab	280.75±0.82b	196.79±5.60b	296.50±1.30c	83.96±4.77bc	29.91±1.79cd	99.71±4.30bc	35.52±1.64de
13	78.80±0.00c	372.88±1.00c	273.50±5.42c	372.92±2.01d	99.38±6.42dc	26.65±1.65c	99.42±1.41bc	26.66±0.31c
25	82.45±0.07d	506.50±2.95d	414.17±2.49d	515.08±3.18e	92.33±13.44c	18.23±2.54b	100.91±7.31bc	19.92±1.33b
50	94.70±0.00e	765.17±2.54e	716.92±3.42e	826.33±0.24f	48.25±2.96a	6.31±1.36a	109.41±3.65c	14.30±0.34a

All data represent the mean of three determinations.

Mean± standard deviation.

Means with the same letter in each column are not significantly different (p<0.05).

Breakdown was measured of the cooked starch to disintegration [13]. Leached amylose is more or less aligned in the direction of flow that contributes to the breakdown [6]. Breakdown value is peak viscosity value minus trough viscosity value. We defined the disintegration rate to measure the disintegration speed of starch paste and its role was better than breakdown value because peak viscosity and trough viscosity were changing at the same time. The disintegration rate is the ratio of breakdown value to peak viscosity value. There was no significant difference in disintegration rate when the concentration of glucose syrup was 1%, 3%, fructose syrup was 1%, 3%, 13% and 25%, and maltose syrup was 1%, 3% and 13%. However the disintegration rate of corn starch decreased when the concentration of glucose syrup was 13%, 25% and 50%, fructose syrup was 50% and maltose syrup was 25% and 50%. It really showed that corn starch in fructose syrup disintegrated more easily than in glucose and maltose syrup. The disintegration rate of corn starch increased in the following order: fructose syrup> maltose syrup> glucose syrup. Molecular corn starch ruptured and had interaction with different syrup resulting in different disintegration rate. At 50% concentration of the three kinds of syrup, the disintegration rate of corn starch decreased. In Table 1 to Table 3, we can see the trough value is closed to the peak viscosity value indicating that disintegration of starch in high concentration syrup was slight when the syrup concentration was 50%. The change may be attributed to the fact that high concentration of syrup reduced the proportion of amylose leaching. Similar results were reported by Ahmed and Williams [19]. Setback value was determined by final viscosity value minus trough viscosity value. The retrogradation rate was defined to indicate how quickly a short-term retrogradation and its role was better than setback value, because final viscosity and trough viscosity were changing at the same time. The retrogradation rate is the ratio of setback to peak viscosity. When the concentration of syrup was low, for instance 1% and 3%, the retrogradation rate of starch showed little change. In addition, increasing syrup concentration to 13%, 25% and 50% resulted in a lower retrogradation rate than the pure starch showing short-term retrogradation of starch was slow in the high concentration syrup solution. There was more or less molecular interaction among sugar, amylose and amylopectin, leading to a decrease in the extent of amylose leaching and it was more difficult to associate directionally, so the retrogradation rate was lower in the high concentration syrup solution.

### Textural Properties of Corn Starch Gels

**Figure 1:**
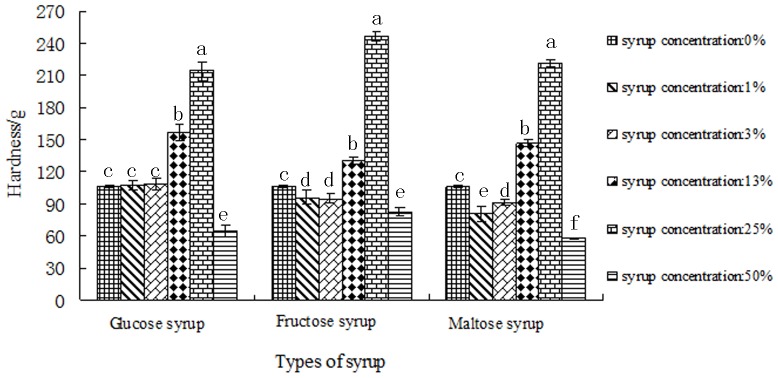
The hardness values of corn starch gels in syrup at different concentrations.

**Figure 2:**
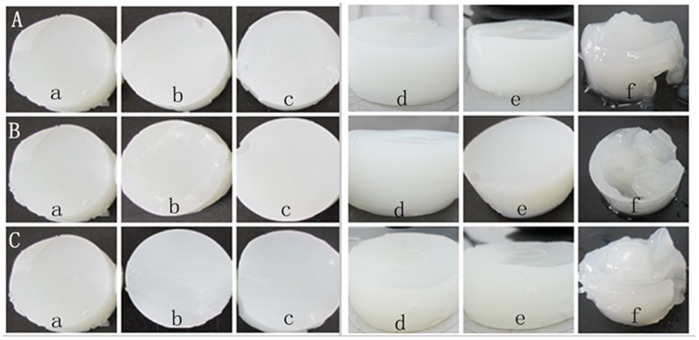
Images of corn starch gels in syrup at different concentrations. A: Corn starch gels in glucose syrup in different concentrations: a (0%), b (1%), c (3%), d (15%), e (25%) and f (50%). B: Corn starch gels in fructose syrup in different concentrations: a (0%), b (1%), c (3%), d (15%), e (25%) and f (50%). C: Corn starch gels in maltose syrup in different concentrations: a (0%), b (1%), c (3%), d (15%), e (25%) and f (50%).

The textural properties of corn starch gels in syrup at different concentrations determined using Texture profile analysis (TPA) tests were shown in Fig. 1 and Table 4. Images of corn starch gels in syrup at different concentrations were shown in Fig. 2.

**Table 4:** The springiness values of corn starch gels in syrup at different concentrations.

Syrup concentrations/%	Glucose syrup	Fructose syrup	Maltose syrup
0	0.99±0.01b	0.99±0.01b	0.99±0.01b
1	0.98±0.00b	0.98±0.03b	0.95±0.00b
3	1.00±0.03b	0.97±0.00b	0.97±0.01b
13	0.98±0.00b	0.95±0.01b	0.96±0.00b
25	0.97±0.00b	0.98±0.01b	0.97±0.02b
50	0.79±0.00a	0.79±0.01a	0.81±0.04a

All data represent the mean of three determinations.

Mean± standard deviation.

Means with the same letter in each column are not significantly different (p<0.05).

### The Hardness of Corn Starch Gels in Glucose, Fructose and Maltose Syrup

Hardness value is the force at maximum compression during first bite [11]. Fig. 1 showed the hardness results of corn starch gels in syrup at different concentrations. It can be seen that when the concentration of syrup was low, the change of hardness values was slight. When concentration of the three kinds of syrup was increased to 13% and 25%, we can see the hardness values of corn starch gels increased dramatically. The highest hardness values were observed when the syrup concentration was 25%. The reason why the hardness increased may be because in syrup-starch system, free water decreased leading to an increase of amylose leaching concentration relatively. Starch molecules were more likely to crash and easier to rank directionally to form three dimensional network structures resulting in an increase in gel hardness. Gunaratne et al. [2] reported that sucrose and glucose increased the hardness of wheat starch gel. They proposed that sucrose and glucose could create more junction zones on amylose chains, thus changed the ordering and intermolecular association of amylose chains, thus resulting in a strong amylose gel matrix network. Gel hardness of wheat starch was increased following the order glucose>sucrose>glycerol. The results indicated the occurrence of starch–polyhydroxy interaction which reinforces the starch granules [2]. There was a particularly low hardness value when the syrup concentration increased to 50%. This was because the syrup concentration was too high to form a powerful gel (Fig. 2).

### The Springiness of Corn Starch Gels in Glucose, Fructose and Maltose Syrup

Springiness value is the distance or length of compression cycle during the second bite [11]. We can see the springiness value increased. From this study, the springiness values of starch gels in glucose, fructose and maltose syrup at different concentrations (0%, 1%, 3%, 13%, and 25%) were similar. The syrup at the concentrations (1%, 3%, 13% and 25%) did not make the springiness of corn starch gels worse (Table 4), suggesting that it had little influence on the corn starch gel’s “rubbery” feeling in the mouth. In Fig. 2, the starch gels all looked very powerful at 0%, 1%, 3%, 15% and 25% concentration. But when the concentration of syrup was 50%, the springiness of starch gel decreased because it could not form a good gel. In Fig. 2, the starch gels looked like viscous and soft sludge separating out a lot of sugar solution.

## Conclusion

The pasting and textural properties of corn starch in glucose, fructose and maltose syrup at different concentrations were observed. The competition between syrup and starch, through association with available water molecules, led to a significant increase in pasting temperature when the concentration of syrup was 13%, 25% and 50%. The peak viscosity, trough viscosity and final viscosity of corn starch in different syrup increased and peak viscosity increased in the following order: fructose syrup> maltose syrup> glucose syrup. Disintegration rate of corn starch increased in the following order: fructose syrup> maltose syrup> glucose syrup. When the concentration of syrup was low, for instance 1% and 3%, the retrogradation rate of starch showed little change. In addition, increasing syrup concentration to 13%, 25% and 50% resulted in a lower retrogradation rate than the pure starch. Increasing the concentration of the three kinds of syrup to 13% and 25%, we can see the hardness of starch gels increased dramatically. There was a particular low hardness value when the syrup concentration increased to 50%. The springiness of starch gels in glucose, fructose and maltose syrup at different concentrations (0%, 1%, 3%, 13%, 25%) were similar.
